# Drug advertising in the German free-of-charge health magazine Apotheken Umschau (Pharmacy review): a critical analysis

**DOI:** 10.1007/s00210-023-02744-7

**Published:** 2023-10-04

**Authors:** Laura Keuper, Roland Seifert

**Affiliations:** Institute of Pharmacology, Hannover Medical School, Carl-Neuberg-Str. 1, D-30655 Hannover, Germany

**Keywords:** Drug advertising, Therapeutic Products Advertising Act (Medicines advertising law), Apotheken Umschau, Pharmacy review, Drug safety COVID-19

## Abstract

In Germany, the Apotheken Umschau (Pharmacy review) is a very popular health magazine for lay people, which is available free of charge in pharmacies. In this study, drug advertising in the Apotheken Umschau was critically analyzed. To our knowledge, studies on drug advertisements in such magazines are scarce. For the analyses, a total of 123 different advertisements from all 48 issues of Apotheken Umschau in 2020 and 2021 were compared. Since 2021 is the first year that was completely characterized by the COVID-19 pandemic, it was thus also possible to work out pandemic effects. More preparations from the categories immune system, eyes and sleep were advertised in 2021. Accordingly, typical complaints home office workers were addressed. Advertisements provided only very modest scientific information, if at all. The Therapeutic Products Advertising Act (Medicines Advertising Law; Heilmittelwerbegesetz (HWG)) provides regulations regarding the handling of drug advertising. However, in various categories, we noted substantial deficiencies of the advertisements to comply to regulations, e.g. the disclosure of adverse effects and omission of the mandatory statement “For risks and adverse effects, read the package insert and ask your doctor or pharmacist (Für Risiken und Nebenwirkungen fragen Sie Ihren Arzt oder Apotheker oder lesen Sie die Packungsbeilage)”. Fifty-one of the 123 advertisements featured females to appeal particularly to this target group. Furthermore, a homogeneous ethnic (Caucasian) picture and a traditional image of German society were presented although this clearly deviates from reality. In conclusion, this study provides insights into the yet poorly studied mechanisms of drug advertisement for lay people. Pharmacological evidence plays a much smaller role than psychology, marketing and traditional societal models. It appears that drug companies intentionally use the “grey area” of the Medicines Advertising Law to optimally promote their products and ignore mandatory statements. This practice must be stopped by stricter legal control to protect the consumer from misinformation. In this way, drug safety will be increased.

**Supplementary Information:**

The online version contains supplementary material available at 10.1007/s00210-023-02744-7.

## Introduction

Apotheken Umschau (Pharmacy review) is a German health magazine that is available free of charge as a customer magazine in 80% of German pharmacies and has been published by Wort and Bild Verlag since 1956. The founder was Rolf Becker, who died in 2014, and the editors-in-chief are currently Dr. Dennis Ballwieser and Julia Rotherbl (https://de.wikipedia.org/wiki/Apotheken_Umschau, last accessed September 28, 2022). The magazine is published twice a month and is aimed at consumers > 40 years of age, to whom topics relating to health are to be conveyed briefly, concisely and in easy-to-understand language. The magazine reaches more than 17 million readers and, thus, has a major impact on consumer behaviours in the pharmacotherapy sector, specifically self-medication (https://de.wikipedia.org/wiki/Apotheken_Umschau, last accessed September 28, 2022).

The “Law on Advertising in the Field of Medicine (Therapeutic Products Advertising Act; Heilmittelwerbegesetz (HWG))” (https://www.anwalt.org/hwg/, last accessed September 28, 2022) or “Medicines Advertising Law” for short came into force in 1965 and established “regulations for dealing with advertising of medical products, medicines or cures” (https://www.anwalt.org/hwg/, last accessed September 28, 2022). It consists of 18 paragraphs and has the overriding goal to protect the consumer. In addition, a differentiation is made between advertising in professional (medical doctor) circles and advertising directed at the public, so-called lay advertising, as in the case of the Apotheken Umschau. In terms of content, both mandatory and prohibited information is regulated.

Sullivan et al. (2020) reported on 1744 US adults regarding the extent to which prescription drug advertising influences doctor-patient interactions. Seventy-six percent reported that they would ask health care providers about advertised medications, and 26% have already done so, of whom 16% received a prescription as a result. Huh and Im (2021) found that patients are often exposed to conflicting drug information due to different forms of advertising. The study concluded that drug advertising has a significant impact on patients’ compliance with medication. In a study on advertisements for selective serotonin reuptake inhibitors, it was noted that a large proportion of the statements in the advertisements are inaccurate and convey an overly positive image of the properties of the drug. In addition, the requirements of the Norwegian Drug Advertising Act are not complied with Spigset (1998). These three examples illustrate the relevance of this issue to pharmacology and the health care system. But overall, there is not much literature on this topic, particularly little internationally published work on the situation in Germany although this is one of the biggest drug markets globally.

Since the Apotheken Umschau enjoys high popularity in Germany, it reaches a large portion of the population and influences their drug consumption behaviour. The aim of this study was to analyze the drug advertisements in this magazine and unmask the mechanisms operating in these advertisements. In addition, the study aimed at assessing the compliance of drug advertisements with the Medicines Advertising Law.

## Material and methods

**Fig. 1:**
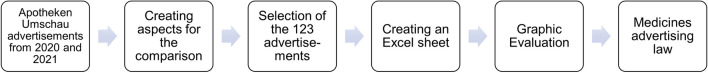
Statistical procedure of the analysis of the advertisements in the Apotheken Umschau, shown in a flow chart

Figure 1 uses a flow chart to illustrate the analysis procedure. In total, all 48 issues of Apotheken Umschau from the years 2020 and 2021 were analyzed. In each case, the first advertisement for the corresponding preparation (drug, product) was documented. Although the products are usually advertised several times, the advertisements differ only minimally from one another. A total of 123 different preparations were considered.

First, Excel tables were created with various parameters that can be adequately compared in a (semi-)quantitative manner. The aspects category of the disease, name, type of preparation (cream, tablet, juice, etc.), manufacturer, indication, effect, stated adverse effects and reports of other readers were compared. It was also analyzed in which respect the preparation name already alludes to the complaints which can be relieved, whether persons, emotions of the persons, persons in movement, body parts, nature motives and the packing are printed. Source, size of the announcement, areas of application of the product, possible warning notes and possible associations of the preparation name with the application purpose were recorded as well. The Excel spreadsheet was revised again after viewing the ads and the 123 selected advertisements were documented. The Excel spreadsheet with the original source data (also containing the actual advertised products) is available from the authors upon reasonable request. In the next step, a graphical evaluation of the data was performed.

An analysis of the Therapeutic Products Advertising Act was performed regarding the implementation of legal requirements in the advertisements. This analysis was carried out with the aid of an Excel sheet that listed mandatory disclosures and prohibitions from the Therapeutic Products Advertising Act (https://www.anwalt.org/hwg/, last accessed September 28, 2022), so that the individual preparations could be entered.

## Results

### Categories and indications of advertisements in 2020 and 2021

**Fig. 2:**
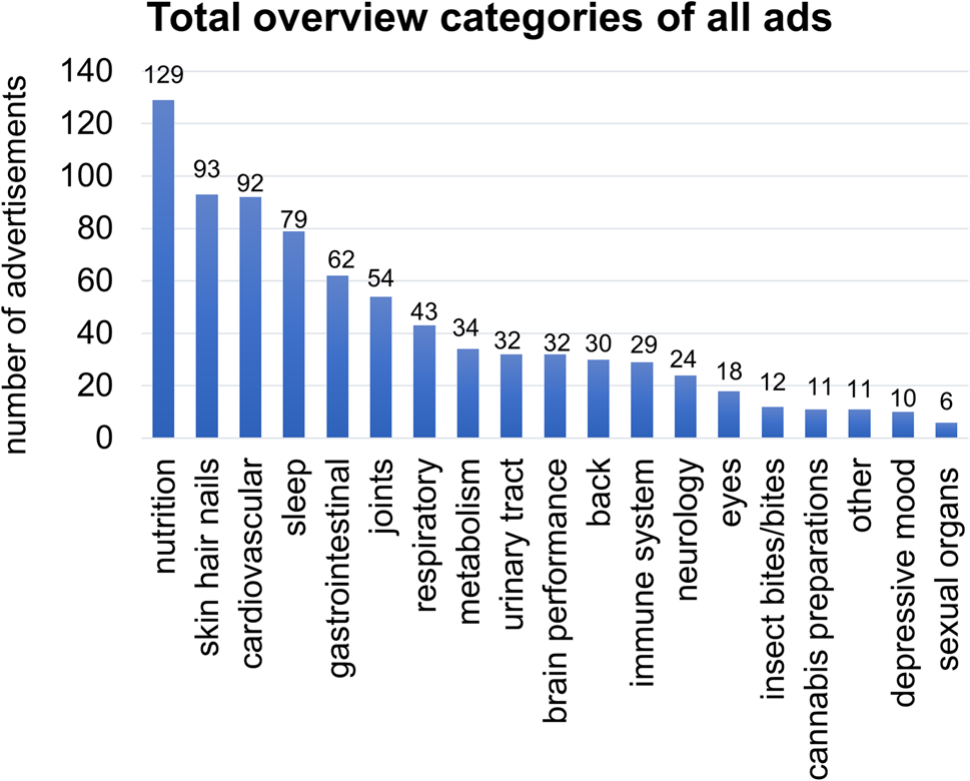
Overall overview of the categories of preparations advertised from 2020 and 2021, shown in a bar chart

Figure 2 presents a total overview of the complaint categories of the respective preparations in the issues of the years 2020 and 2021. The frequency with which the preparations were advertised was considered, so that a total of 801 advertisements are included in the overview. One hundred twenty-nine advertisements promoted preparations from the category nutrition, 93 focused on the category skin hair nails, 92 on cardiovascular, 79 on sleep, 62 on gastrointestinal and 54 on the category joints. The categories cannabis preparations and other were advertised with 11 ads each, depressive mood with 10 and sexual organs with 6 ads.

**Fig. 3:**
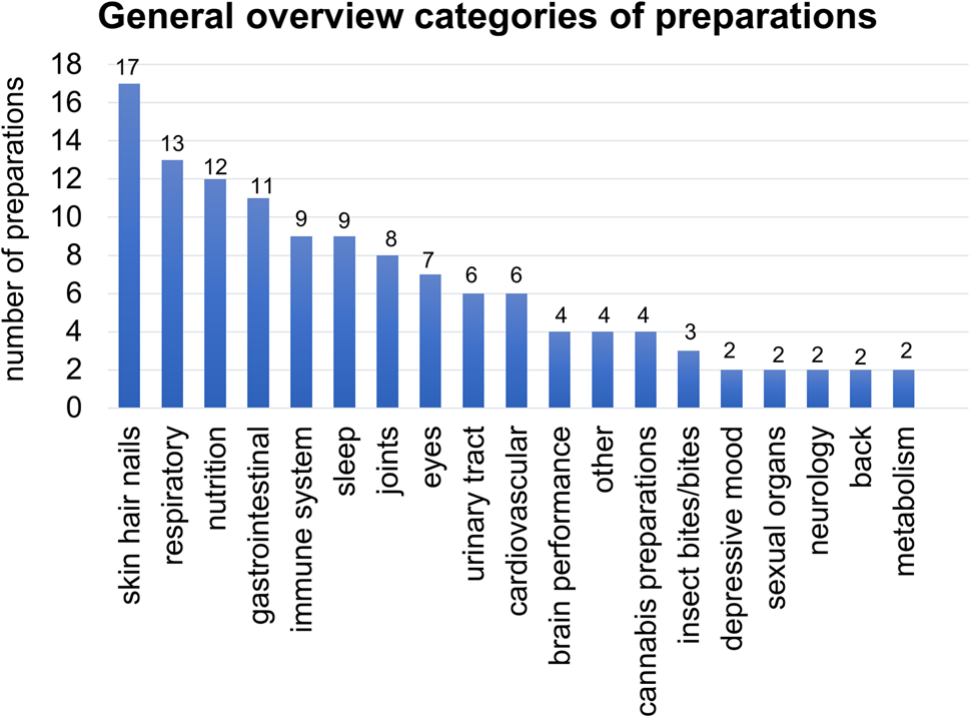
Overall overview of the categories of the 123 different preparations, shown in a bar chart

In Fig. 3, the categories of the 123 different preparations are shown without considering how frequently the individual preparations were advertised in each case. Seventeen of 123 preparations can be classified in the category skin, hair and nails, 13 in the category respiratory tract, 12 in the category nutrition, 11 in the category gastrointestinal tract, 9 each in the categories immune system and sleep, 8 in the category joints, 7 in the category eyes and 6 each in the categories urinary tract and cardiovascular system, whereas the category cannabis preparations was represented by four preparations and the categories metabolism, back, neurology, metabolic disorders and depressive mood by two preparations each. Overall, the different representations in Figs. 2 and 3 are similar with respect to the frequency of the categories.

**Fig. 4:**
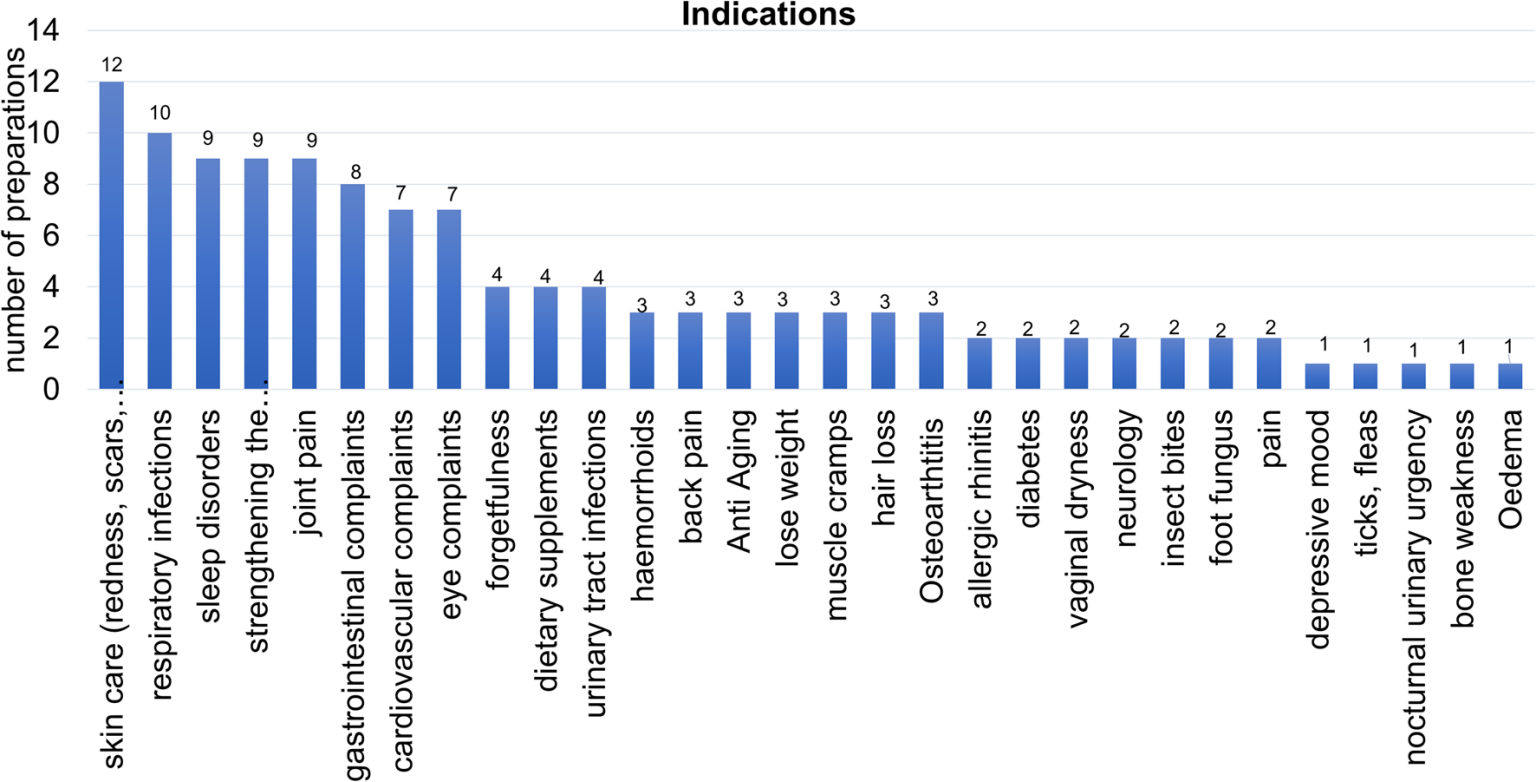
Overview of the indications of the 123 preparations, shown in a bar chart

The categories were subdivided once again more precisely according to individual indications (Fig. 4). The aspect of skin care (redness, scars, wounds, etc.) was advertised in 12 of the 123 ads, respiratory infections represented an indication in 10 ads, the preparations from the categories of sleep disorders, strengthening the immune system and joint pain were advertised in 9 ads each, 8 ads of the preparations addressed gastrointestinal complaints, 7 each cardiovascular and eye complaints and 4 forgetfulness, dietary supplements and urinary tract infections. Several other indications were advertised three times or less often.

**Fig. 5:**
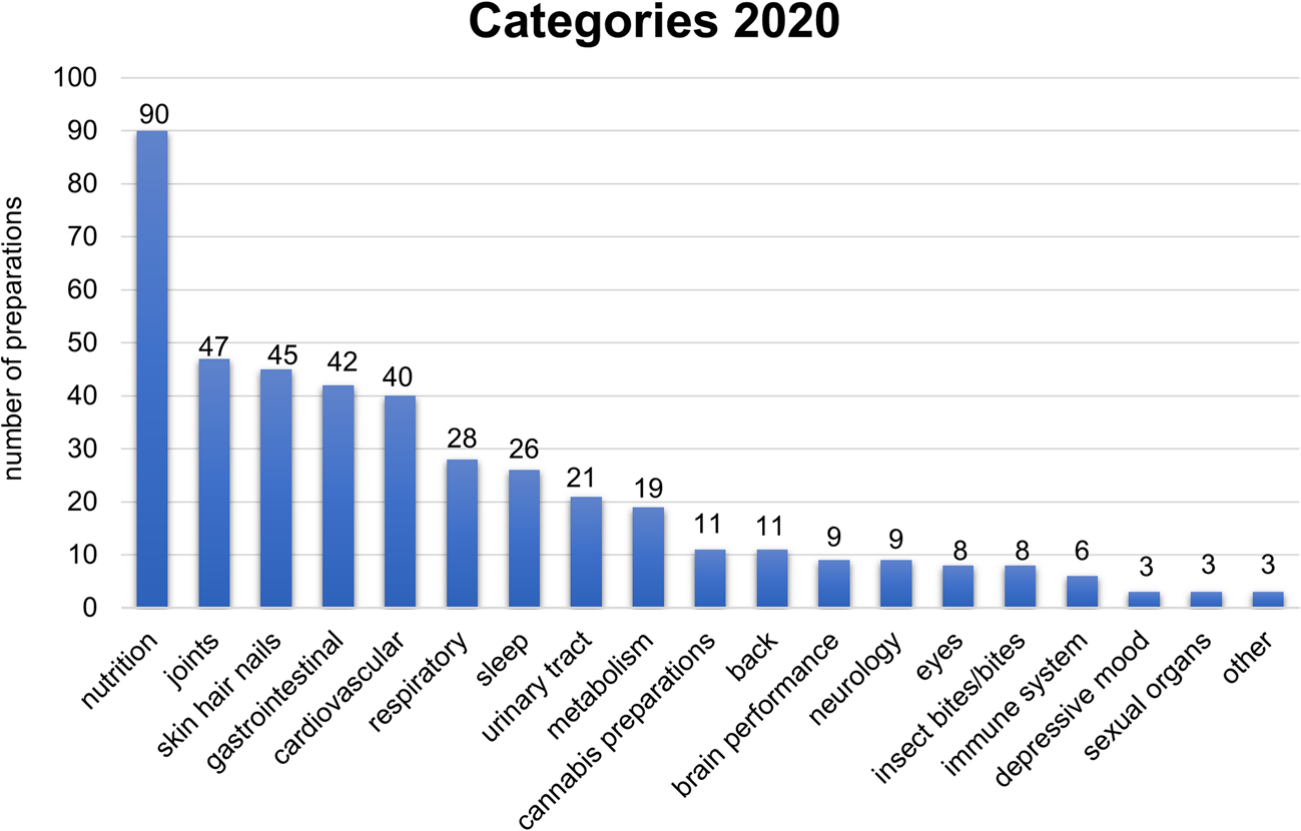
Overview of the categories in 2020 advertisements; shown in a bar chart are the numbers of all advertisements of the preparations

In 2020, 90 advertisements of the preparations were assigned to nutrition, 47 adevertisements can be assigned into the joints category, 45 into the skin hair nails category, 42 into the gastrointestinal category, 40 into the cardiovascular category, 28 into the respiratory category and 26 into the sleep category. In addition, 11 advertisements each were assigned to the cannabis preparations and back categories, 9 to the categories brain performance and neurology, 8 to the categories eyes, insect bites/bites, 6 to the category immune system and 3 each to the categories depressive mood, sexual organs and other (Fig. 5).

**Fig. 6:**
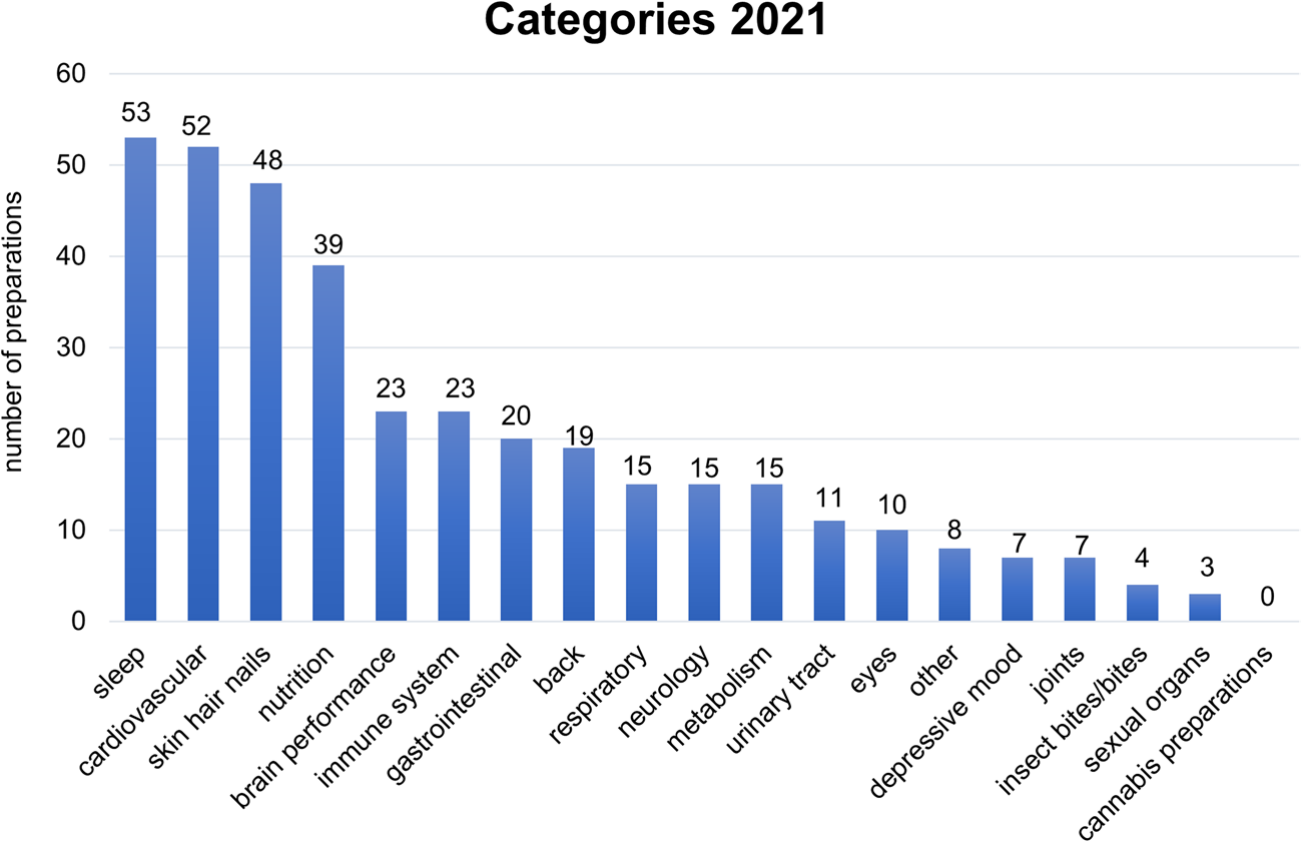
Overview of the categories in 2021 advertisements; shown in a bar chart are the numbers of all advertisements of the preparations

Furthermore, an overview of the categories of the advertisements of all preparations in the 2021 issues was prepared. Fifty-three advertisements can be assigned to the category sleep, 52 to the category cardiovascular, 48 to the category skin hair nails, 39 to the category nutrition, 23 each to the categories brain performance and immune system, 20 to the category gastrointestinal, 19 to the category back, 15 each to the categories respiratory tract, neurology and metabolism, 11 to the category urinary tract, 10 to the category eyes, 8 to the category other and 7 each to the categories depressive upset and joints. The insect sting/bite category was advertised 4 times in 2021, the reproductive organs category 3 times and the cannabis preparations category not at all (Fig. 6).

### Scientific validation of advertisements

**Fig. 7:**
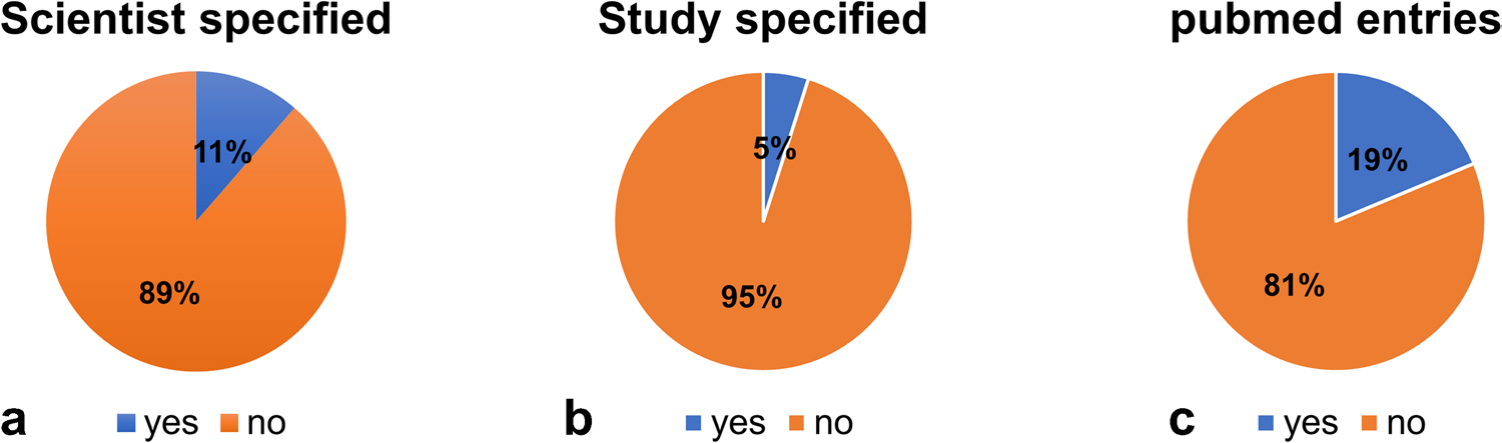
Scientific validity of the advertisements; **a** specification of concrete scientists; **b** specification of concrete studies; **c** search for PubMed entries on the preparations. One hundred twenty-three preparations were analyzed

**Fig. 8:**
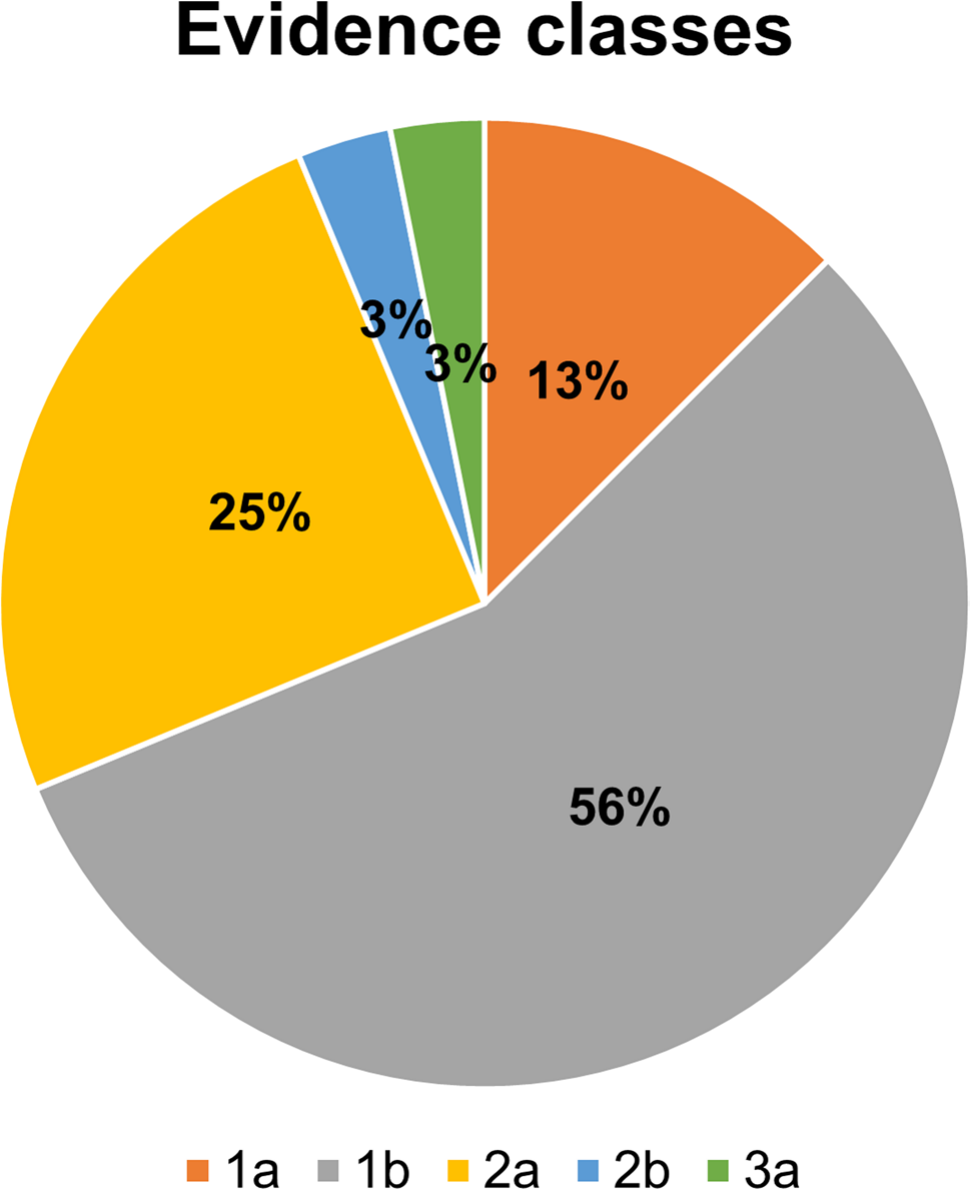
Evidence classes of the PubMed studies. The numbers refer to the 19% of “yes” drugs with PubMed entries (a total of 23 drugs) in Fig. 7c

An important focus of our analyses was the extent to which the content in the advertisements is scientifically based. In 11% of the ads for the 123 preparations, individual scientists were mentioned by name (Fig. 7a). In just 5% of the analyzed preparations, specific scientific studies were indicated (Fig. 7b). In addition, it was documented whether entries for the individual preparations could be found in the PubMed database. This was the case for only 19% (a total of 23 out of 123) of the preparations (Fig. 7c). Only clinical studies that investigated drug efficacy in patients were considered. The clinical studies for the remaining 23 preparations with PubMed entries were divided into different evidence classes to obtain a more precise impression regarding their credibility. For this purpose, the classification of the evidence classes from Deutsches Ärzteblatt (German Medical Journal) was used (Hörle and Kroll 2005). Class 1a includes the studies that, according to PubMed, have demonstrated evidence through meta-analyses of multiple randomized controlled trials and class 1b includes studies that have demonstrated evidence through at least one randomized controlled trial. Class 2a refers to those studies that are reported to have demonstrated evidence in at least one randomized controlled trial. In class 3a, the evidence is based on well-designed non-experimental studies or a review of case-control studies, in class 3b, on single-control studies, in class 4, on case reports or studies with methodological flaws and in class 5, on reports by experts and/or clinical experiences of recognized authorities. In total, studies for four preparations could be classified in category 1a, for 17 in evidence class 1b, for 8 in evidence class 2a and for one in class 3a (Fig. 8). In several studies, the respective manufacturer had financially supported the investigations. The conflict of interest could evidently affect the outcome of the studies. We did not analyze the actual scientific validity of the relevant studies within the scope of this study.

### Formal analysis of the advertisements

Fifty-one of the 123 different ads depict females. In 39 ads, no persons are depicted, in 16 spouses or partners, in 6 families and in just 5 ads males. In 3 advertisements, twins and additionally a child are printed, in 2 advertisements females well-known in Germany (Rosi Mittermaier and Marie-Luise Marjan) and in just one advertisement two women are depicted (Figure S 1). It is noticeable that only heterosexual couples are printed, thus creating a classic image of a traditional family. In 60% of the advertisements, the printed persons appear to be happy. In 33% of the ads, no emotions are depicted, in 5% the people appear in pain, and in 2% of the ads, one part of the printed people seems happy and the other part also in pain. People in motion are shown in 20% of the ads (Figure S 2). Thirty-four percent of individuals appear younger than age 40, 31% appear to be over age 60, 27% appear older than age 40 and 8% are children (Fig. S 3). Regarding the ethnicity of the persons shown, 100% are Caucasian (Figure S 4). In 22% of the ads, the focus is on a body part due to specific complaints that the preparation is supposed to alleviate (Figure S 5). Nature motifs are printed in 39% of ads (Figure S 6).

In 87% of all advertisements, the product name already provides suggestions of the complaints or the disease that the preparation is intended to alleviate. Twelve percent of the product names are chosen neutrally and do not allow any associations, and in just one product an actual INN name, the “International Nonproprietary Name” (https://www.who.int/teams/health-product-and-policy-standards/inn/, last accessed September 28, 2023) is provided (Figure S 7). In 99% of the advertisements, the package of the product or the tablets themselves are printed (Figure S 8). In 3% of the ads, there are thematically appropriate texts from the editorial team alongside the actual advertising. This is not the case in 97% of the ads (Figure S 9).

In 27% of the advertisements, the text colour white was used and in 26% the colour black. Furthermore, the colour blue was chosen in 16% of the advertisements, the colour red in 13% and the colour green in 10%. Orange was used in 5% of the ads, purple in 2% and yellow in 1% of the analyzed ads (Figure S 10).

In 64% of the advertisements, mandatory statements, such as adverse effects and ingredients, are printed in smaller font than the actual advertising claims. In 33% of the ads, the mandatory statements are the same size, and in 3% of the ads, they are only slightly smaller than the advertising statements (Figure S 11a). In 84% of the advertisements, the mandatory text was easy to read but this is a subjective assessment (Figure S 11b). Sixty-five percent of the mandatory information was printed in black, 28% in white and 7% in blue (Figure S 11c).

### Analysis of the advertisements with respect to the Therapeutic Products Advertising Act

Table 1 provides an overview of how well the requirements set out in the Therapeutic Products Advertising Act are complied with (https://www.anwalt.org/hwg/, last accessed September 28, 2022). Figures with a green font represent aspects that are reliably complied with, figures with a yellow font represent aspects for which it was not possible to determine with certainty whether the regulations were complied with and figures with a red font illustrate specifications that were not complied with. The column on the left shows the analyzed criteria.

**Table 1:**
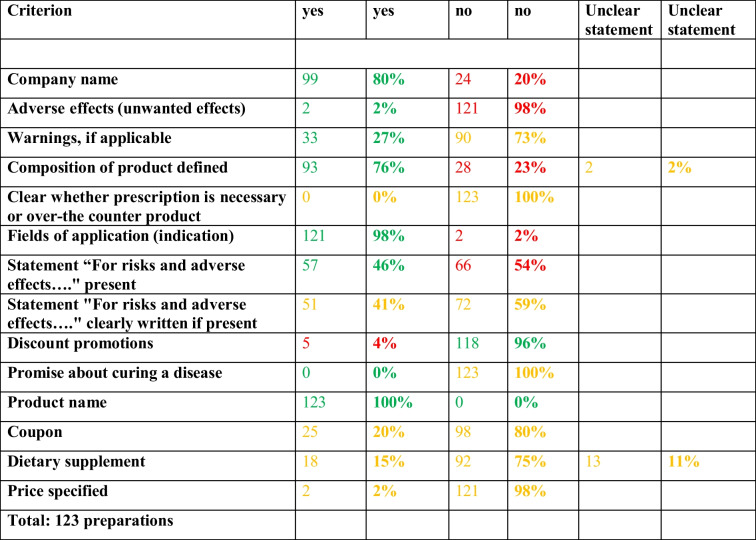
Compliance with the Therapeutic Products Advertising Act, presented in a table. Absolute numbers and percentages are shown for each parameter. Green, compliance with legal regulations. Red, non-compliance with legal regulations. Yellow, ambiguous compliance with legal regulations

The name of the company is a mandatory information, which is printed in 80% of the advertisements. Adverse drug reactions are indicated in just 2% of the advertisements, warnings in only 27% of the advertisements and the exact composition of the product is printed in a mere 76% of the advertisements. In 2% of the advertisements, the exact composition of the product remains unclear if imprecise information such as “medicinal plants” is given. In such cases, yellow was chosen as the colour. Prescription drugs may only be advertised in professional circles, but it remained unclear in all cases whether the preparations advertised are truly over-the-counter products or not.

The indications (clinical uses) of the products are evident in 98% of the advertisements. The statement “For risks and adverse effects, read the package insert and ask your doctor or pharmacist (Für Risiken und Nebenwirkungen fragen Sie Ihren Arzt oder Apotheker oder lesen Sie die Packungsbeilage)”, as already shown above, is a mandatory statement. This is printed in just 46% of the advertisements, surprisingly. It was also studied to which extent this statement, as required by law, can be clearly distinguished from the actual advertising statements. In just 41% of the advertisements with the statement, a clear demarcation was possible. However, the yellow colour underlines the subjective influence. Discount promotions whose total value exceeds 1€ are not permitted by law. But in 4% of the ads, such promotions were made. Lastly, it is forbidden to make promises to the readers that the preparation will cure the disease. Such concrete statements were not documented, but in every advertisement a certain effect is suggested to the reader and promised in some way.

The product name was presented in all the advertisements analyzed. In addition, a coupon was printed in 20% of the advertisements, which can be cut out and handed to the pharmacists to purchase the preparation quickly and easily. Furthermore, in 15% of the advertisements, a label indicating that the product is a dietary supplement was found. In 75%, the products are apparently herbal drugs. In 11% of the advertisements the classification remained unclear. The aspects coupon and dietary supplement are ambiguous within the Therapeutic Products Advertising Act. Almost all advertisements also remained unclear about pricing of packages and daily defined dose costs (DDD costs) which is the gold standard in all pharmacoeconomic analyses (Ludwig et al. 2023; Trabert and Seifert 2023). Along this avenue, no comparisons with costs of alternative therapies were provided.

**Fig. 9:**
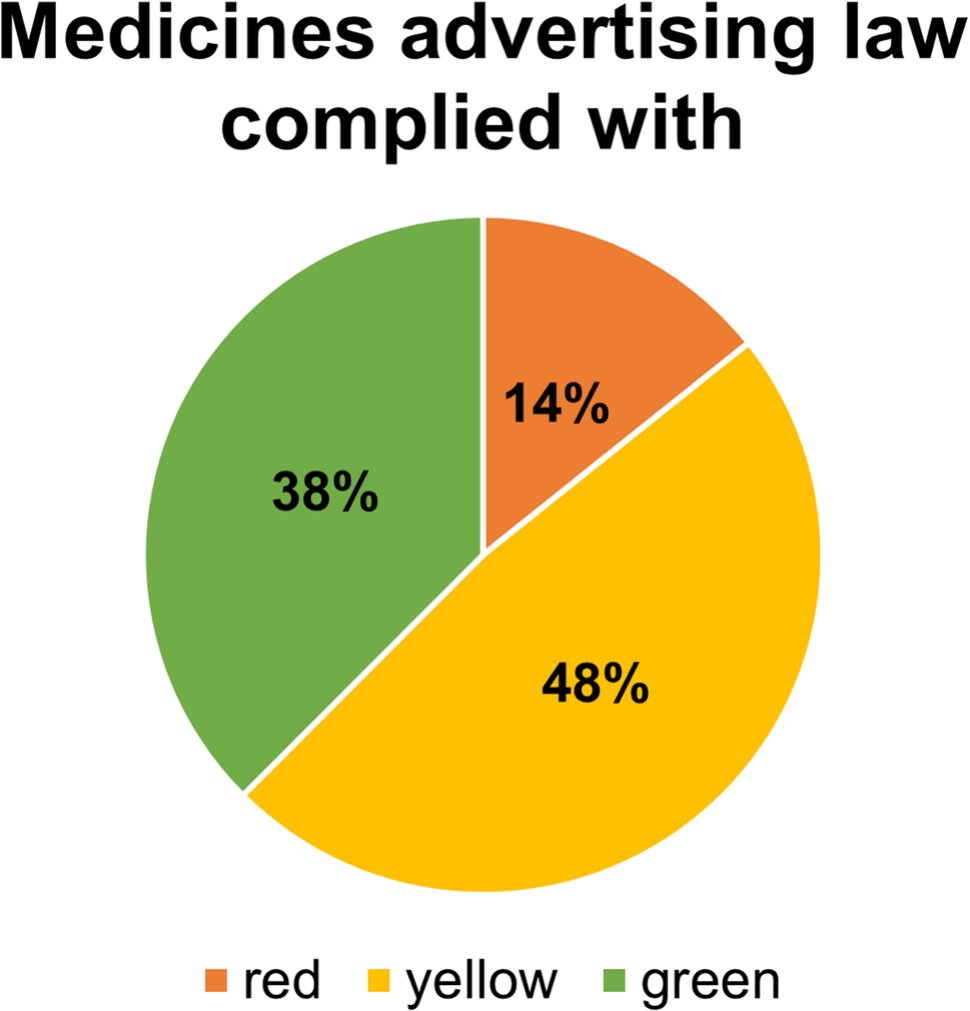
Overview of compliance of mandatory disclosures with the Therapeutic Products Advertising Act in advertisements, shown in a pie chart. Data were derived from Table 1

Table 1 was then presented again with the aid of a pie chart. For 48% of the analyzed mandatory disclosures or prohibitions, it remained ambiguous to what extent the legal requirements were complied with. In 38% the mandatory disclosures analyzed were complied with and in 14% they were not complied with (Fig. 9).

## Discussion

### The pharmacoeconomic dimension

Apotheken Umschau addresses consumers who invest into their health by purchasing over-counter products in pharmacies. Based on the analysis of the advertisements, it can be assumed that affluent middle-aged and older persons are the main target group. Older people may be forgetful, but for this clientele, product coupons are provided in 20% of the advertisements so that the customers do not have to remember the product name.

Health may be perceived so important by this group that the actual price for the products is not so important for them. In fact, except for offering rebates in several cases (Table 1), it remained completely elusive for most products how expensive they are. The daily defined dose (DDD) costs, the gold standard in pharmacoeconomics, remained undisclosed in all cases (Trabert and Seifert 2023; Ludwig et al. 2023). The concept of DDD costs was not mentioned in any of the advertisements. As a result of this non-information, the consumer cannot make his/her own calculations of therapy costs, although the mathematics behind this concept is simple. Thus, for pricing, the advertisements did not provide access to information that is important for every consumer. In all other areas of daily life, price analysis and comparison are a must, but apparently not so in the drug market. Thus, most unfortunately, based on the available data, we cannot present a pharmacoeconomic analysis of the products. The DDD costs of the advertised products must be analyzed in future studies. This is not a trivial task because prices may differ among various types of pharmacies, particularly online pharmacies, and differ depending on the package size.

### Complaint categories and indications

From the overview of the categories of preparations from the 2020 and 2021 issues, the nutrition category is advertised most frequently across both years (Fig. 2). This result implies that nutrition and weight loss have high priority in German society. Especially at the beginning of the year, this category was documented regularly, which suggests that the Apotheken Umschau is reacting to the fact that many people are considering New Year’s resolutions to lose weight. A survey commissioned by DAK-Gesundheit showed that this was a resolution for 61% of respondents in December 2022 (https://www.dak.de/dak/bundesthemen/gute-vorsaetze-2023-2594612.html#/, last accessed April 22, 2023).

The categories skin hair nails, cardiovascular and sleep are also frequently advertised. In contrast, joint complaints only appear in sixth place (Fig. 2), although these represent a major burden, especially with increasing age (Endres 2021) https://alley.de/magazin/gelenkschmerzen-ursache-verlauf-tipps/, last accessed April 21, 2023). Possibly, this finding results from the fact that drug companies know, for example based on the *Arzneiverordnungsreport* (AVR, *Drug prescription Report*) that inexpensive drugs, such as paracetamol, metamizole or ibuprofen, are effective alternatives (Ludwig et al. 2023).

The analysis of the indications underscores that the categories skin care, respiratory infections, sleep disorders and joint complaints are important. In addition, the great importance of preparations intended to strengthen the immune system is evident (Fig. 4). During the COVID-19 pandemic, preparations supposedly strengthening the immune system were heavily advertised. One of “hyped” drugs was vitamin D, and doctor prescriptions in Germany increased sharply (*Arzneiverordnungsreport* (AVR, *Drug prescription Report*) from 2022 (Ludwig et al. 2023)). However, there is no proven beneficial effect of vitamin D in COVID patients (Bignardi et al. 2023). This example illustrates that, because of advertising, the population is willing to take preparations whose efficacy has not yet been proven, without thinking about potentially negative effects (Taylor and Davies 2018).

### Impact of the COVID-19 pandemic

The year 2020 was partially affected by the COVID-19 pandemic, and the year 2021 was completely dominated by the COVID-19 pandemic. Thus, our analysis of Apotheken Umschau from 2020 and 2021 provided a unique opportunity to obtain novel insights into drug marketing mechanisms. Important differences emerged when comparing the 2020 and 2021 categories of advertisements. In 2020, the nutrition category was the leader (Fig. 4). In 2021, the category most frequently advertised was sleep, which only ranked seventh in 2020. More advertisements from the categories immune system (3 in 2020; 23 in 2021), back pain (11 in 2020; 19 in 2021), eyes (8 in 2020; 10 in 2021) and depressive mood (3 in 2020; 7 in 2021) were recorded in 2021. In contrast, in 2021, gastrointestinal advertisements (20) played a much smaller role than in 2020 (42).

Due to the general mask requirement during the COVID-19 pandemic, fewer gastrointestinal infections were spread, whereas back pain and dry eyes represent typical complaints of home office workers (Hallman et al. 2021). Various studies have examined the extent to which working from home has an impact on the prevalence of musculoskeletal complaints. In particular, back and neck pain depends on the office chairs and monitors used, and these conditions are likely to be suboptimal in the home office in most cases (Chim and Chen 2023). In addition, a study on university students who were required to attend online classes during the pandemic found that of 1450 students surveyed, 42.8% suffered from mild/moderate dry eye symptoms and 34.7% suffered from severe symptoms (Cartes et al. 2021). In contrast, more time could be invested in sufficient sleep, raising the awareness for this part of life (Hallman et al. 2021). Accordingly, advertisements for sleeping aids rose from 26 in 2020 to 53 in 2021. Thus, drug advertisements in Apotheken Umschau in 2021 rapidly picked up actual and perceived health issues related to the COVID-19 pandemic. The tools by which drug companies identify changing market demands probably entail customer questionnaires.

### Scientific basis of the advertisement content

Apotheken Umschau emphasizes the high importance of evidence-based medicine on its website (https://www.apotheken-umschau.de/ueber-uns/, last accessed August 15, 2023). Specific scientists are mentioned by name in 11% of the advertised preparations (Fig. 7a). In addition, scientifically inaccurate statements such as “According to studies” and “Based on my father’s research” are made with no references. Specific scientific studies are cited in only 5% of the analyzed products (Fig. 7b). In addition, the PubMed database was searched for entries of the products independently of the advertisements themselves. This search showed that entries are available for just 19% of the 123 preparations (Fig. 7c). From the clinical studies on the 23 preparations identified in PubMed, 13% could be classified as evidence class 1a, 56% as evidence class 1b, 25% as evidence class 2a and 3% each in class 2b and 3a (Fig. 8). In addition to the fact that there are no entries at all for the vast majority of preparations in the PubMed database, it is noticeable that only exceedingly few studies present evidence by meta-analyses of several randomized, controlled studies. Accordingly, it is difficult for the authors to comprehend the declared scientifically based content of the magazine. But for a lay person, it is impossible to assess the validity of statements on the topic in Apotheken Umschau because they generally do not know the accepted scientific standards. In real science, statements such as “According to studies” and “Based on my father’s research” are not only worthless but irreversibly destroy the reputation of any serious scientist. Peer-reviewed studies in established scientific journals are the gold standard.

### Suggestive elements in advertisements

Regarding the persons depicted in the advertisements, single females are presented in particular (Figure S 1). This suggests that the majority of Apotheken Umschau’s clientele are female readers and that they can therefore identify better with the advertisement. The Eucerin Women’s Study 2021 (https://www.eucerin.de/specials/frauenstudie-2021, last accessed May 14, 2023), which included women from different age groups and life phases, showed that pharmaceutical companies are aware of this aspect. This study showed that the comparison among women is strongly age-dependent. While young women compare themselves with each other in a competitive fashion (to assess their attractiveness and market value), older women have predominantly left this phase behind (https://www.eucerin.de/specials/frauenstudie-2021, last accessed May 14, 2023). These results fit in with the fact that the Apotheken Umschau is particularly aimed at women aged 40 and over and features images of single women, eliminating potentially negative factors for drug marketing such as female-female comparison and competition. In addition, couples or families convey a harmonious image. Moreover, when people are shown, they appear to be happy in most advertisements (Figure S 2), which underlines the notion that a positive atmosphere for drug sales should be created by advertisements. Overall, a classic conservative image of the traditional family is created, a value system supposedly preferred by older people (i.e. the target group (readers) of Apotheken Umschau). Potentially marketing-disruptive elements such as persons with migratory background or homosexuality are avoided in the drug advertisements (see also below).

About one-third of the persons depicted seem to be younger than 40 years of age, a third between 40 and 60 years and a third above 60 years (Figure S 4a). Thus, a broad age spectrum is represented, so that as many readers as possible can identify themselves with the advertisements. This strategy is successful since Apotheken Umschau reaches more than 17 million readers. Children are represented much less frequently, just at 8% because they are not an interesting target group for high drug sale volumes. Strikingly, 100% of the persons depicted appear to be Caucasian and accordingly create the image of an ethnically homogeneous society in Germany (Figure S 4b). However, according to the microcensus, 26% of the people living in Germany had a migration background in 2019 (https://www.bamf.de/DE/Themen/Forschung/Veroeffentlichungen/Migrationsbericht2019/PersonenMigrationshintergrund/personenmigrationshintergrund-node.html#:~:text=2019%20had%20according%20to%20figures%20from%20the%20Microcensus%2021%2C2%20million,share%20of%20foreign%C3%A4ndish%20citizens%C3%B6rigen%20betr%C3%A4gt%20damit%2047%2C6%20percent, last accessed March 19th, 2023), and ethnic heterogeneity actually increases. Thus, advertisements in Apotheken Umschau create a picture of the German society that is very far from reality and excludes a large fraction of the population. Possibly, persons with a migration background are deemed to be less affluent and, therefore, a less attractive clientele for the advertised products.

It was assumed that nature motifs would be integrated into numerous advertisements, as forests and nature represent a place of retreat and relaxation for many people and could therefore create a pleasant atmosphere. Viewing nature motifs has a positive effect on recovery from psychological stress (Brown et al. 2013) and improves cognitive performance, which could also be used during work, for example (Daniels et al. 2021). This aspect was only partially confirmed in the analyses. Nature motifs are printed in only 39% of the ads (Figure S 6). Perhaps, the advertisements for drugs in Apotheken Umschau intend to differentiate themselves from potentially competing “wellness products”. A specific body part due to ailments that the drug is supposed to alleviate is the focus in just 22% of the ads (Figure S 5). Evidently, advertisements do not intend to be too negative by pointing to a dysfunctional organ. Rather, positivity in advertisements may enhance drug sales.

We also analyzed to which extent the name of the preparation is suggestive and alludes to complaints that are to be alleviated. This could increase confidence in the effectiveness of the preparation and augment a placebo effect (Pardo-Cabello et al. 2022). In 87% of the advertisements, the preparation name provided suggestive clues to the complaints or diseases that the preparation was intended to alleviate. Twelve percent of the names were chosen neutrally and only one name was the actual International Nonproprietary Name (INN, World Health Organization) (https://www.who.int/teams/health-product-and-policy-standards/inn/, last accessed September 28, 2022) (Figure S 7). But in professional pharmacotherapy, the INN are the gold standard for communication and prescription, not trade names of products.

In 99% of the advertisements, the package of the preparation or the tablets themselves are shown (Figure S 8). In this way, the recognition value of the products was increased. In 3% of the advertisements, thematically appropriate texts from the editorial team of Apotheken Umschau were printed alongside the advertising (Fig. S 9). This enables the reader to “delve” more deeply into a topic and obtain more information. This small number of links between articles and advertisements indicates that most advertisements are placed individually by the respective drug company.

It became apparent that the colour white for text plays an important role with 27%. Black (26%), blue (16%), red (13%) and green (10%) are also frequently used. The colours orange (5%), purple (2%) and yellow (1%), on the other hand, are chosen less frequently (Fig. S 10). Overall, a wide range of different colours was used, but most of the ads are printed in black and white, so that these are the dominant colours and a rather plain colour image is created. Pharmaceutical companies are aware of the effect of colours, so that a sleeping pill, for example, should be white or blue, as it works better for reasons of colour psychology. The colour blue creates a relaxing and calming atmosphere. Products for stomach complaints are preferably depicted in green, and strong painkillers and preparations from the cardiovascular category are red (https://www.hexal.de/hcp/magazin/antwortgeber-magazin/wirkfaktor-mensch, last accessed April 23, 2023). In addition, colours can “set positive impulses in patients” (Evans 2017). Future systematic clinical studies will have to examine to which extent different colours affect drug efficacy, via a placebo or nocebo effect. This constitutes a largely unchartered research area (Pardo-Cabello et al. 2022).

### Insufficient compliance with the Therapeutic Products Advertising Act

Based on the Therapeutic Products Advertising Act, analyses were carried out to determine the extent to which the legal requirements are complied with using the example of the Apotheken Umschau. The indication of the name of the company in the advertisement is a mandatory information. This is implemented in 80% of the ads. Possibly, the remaining 20% prefer not to disclose their identity to avoid communication with the public. The indication of adverse effects is also a mandatory information according to the German Drug Advertising Act. But this critical issue is considered only in 2% of the advertisements. Also from the pharmacological perspective regarding drug and consumer safety, this is inacceptable. Furthermore, possible warnings must be printed, which is the case in just 27% of the advertisements. In addition, the exact composition of the preparation must be documented. The ingredients are stated in a mere 76% of the advertisements. It also remains unclear whether the advertised drugs are available over-the-counter or via prescription only.

The statement “For risks and adverse effects, read the package insert and ask your doctor or pharmacist” must also be included by law in every advertisement in lay advertising. This aspect is complied only in 46% of the advertisements. Furthermore, according to the Therapeutic Products Advertising Act, this sentence must be clearly distinguishable from the other advertising statements. Although this is a subjective finding, the analyses showed that this was the case in only 41% of the advertisements. Most of the mandatory information is printed in a rather small font size, whereby it must be kept in mind that the Apotheken Umschau is addressed to consumers over 40 years of age. This will decrease the likelihood that older people will and can read this important text. Thus, font size has probably a major impact on which information reaches the consumer. Too small font size is also a major problem in package inserts from prescription drugs (Arning and Seifert 2023).

In addition, the law states that discount promotions are prohibited. But in 4% of the advertisements, these are nevertheless advertised, so that, for example, discount coupons are printed or giveaways are promised to entice the reader to purchase a product. But price transparency of products is not provided in most advertisements (Table 1). It is not permitted to promise potential buyers specific effects of the preparations or to suggest to them that the disease or symptoms will worsen if they are not taken. Although specific promises are not observed, this aspect is also a subjective assessment, as almost every advertisement suggests a certain effect to the reader.

According to the Therapeutic Products Advertising Act, the indication of the product name is also a mandatory information, which is considered in each of the 123 advertisements. Evidently, this is in the interest of the drug company as well because without product name (trade name) there is no business. But often it remained unclear whether a product is a drug or a dietary supplement. However, this is very important point because the legal requirement for drugs and dietary supplements are very different (Trabert and Seifert 2023).

A study from Arzneimittelbrief (https://der-arzneimittelbrief.com/artikel/1999/das-heilmittelwerbegesetz-und-der-verbraucherschutz, last accessed September 28, 2022) showed that there were already significant problems regarding compliance of drug advertisement with the legal requirements in 1999. The experts agreed that there were legal uncertainties (https://der-arzneimittelbrief.com/artikel/1999/das-heilmittelwerbegesetz-und-der-verbraucherschutz, last accessed September 28, 2022). From the data of the present study, it can be concluded that the situation, legally, scientifically and practically, has not changed much in almost 25 years. Drug advertisement has a major impact on prescription behaviour of physicians (Kalb 2004). Therefore, it must be assumed that advertisement in the Apotheken Umschau must also have an impact on the sales numbers of the advertised products, but these numbers are not known to us.

Most importantly, the modern principles of evidence-based medicine are mostly not adhered to in advertisements. According to the Therapeutic Products Advertising Act, for example, it is prohibited to advertise ineffective preparations. However, for most preparations advertised in Apotheken Umschau, there is no scientific evidence for effectiveness at all (Figs. 7 and 8). Thus, advertisement should not be allowed from a legal perspective. Huh and Im (2021) reported that patients’ compliance about drug intake depends to a large extent on the advertised positive effects or the adverse effects presented. This most likely represents a placebo effect (Pardo-Cabello et al. 2022). Thus, a placebo effect may convey the impression to the public (and federal authorities) that the advertised preparations “work”. Thus, drug companies appear to use the placebo effect to support assumed drug effectiveness.

In aggregate, there are major scientific quality deficits and legal deficits in drug advertisements published in Apotheken Umschau. Marketing and psychological considerations clearly dominate pharmacological considerations and the principles of evidence-based medicine. And even of more concern, it is the impression that drug companies exploit the ambiguous wording (grey area) of the Therapeutic Products Advertising Act at their advantage to optimally promote their products. This is not a new behaviour but had already been observed 25 years ago (https://der-arzneimittelbrief.com/artikel/1999/das-heilmittelwerbegesetz-und-der-verbraucherschutz, last accessed September 28, 2022), with little change in the meantime. The missing shift of advertisements towards evidence-based medicine principles is due to the lack of legal controls from federal institutions in Germany. Only a law that is enforced is an effective law. If there is no enforcement, escape mechanisms evolve to circumvent regulations as is evident from the large yellow area in our analysis (Table 1 and Fig. 9). Magazines depending on advertisements for covering the publication costs have a classic conflict of interest because strict enforcement of legal requirements will inevitably reduce the number of advertisements and/or reduce the market impact of “legally more correct” advertisements and, thereby, jeopardize the future existence of the magazine. Thus, such magazines may also have an interest to exploit grey legal areas or at least not to counteract grey area actions of drug companies.

This study has revealed several parameters that can be easily used by legal authorities to check for adherence to legal requirements. Checking for statements on adverse effects is an excellent starting point, but it requires manual screening of advertisements. But simple automated text screening of advertisements for the presence of the sentence “Für Risiken und Nebenwirkungen fragen Sie Ihren Arzt oder Apotheker oder lesen Sie die Packungsbeilage” will reveal that many product advertisements do not comply to fundamental legal requirements (Table 1).

### Limitations of the study

Despite the care taken in conducting the analyses, there are some limiting factors. We used only publicly available information and had no access to proprietary knowledge. Various aspects, in particular the investigation of compliance with the Therapeutic Products Advertising Act, are difficult due to the imprecisely worded text. The current analysis was not performed by lawyers, but was carried out independently by authors with medical and pharmacological training to the best of their knowledge and understanding. In addition, graphic aspects, such as whether advertising statements are clearly delineated, are somewhat subjective. The authors are neither psychologists nor marketing experts, limiting the depth of the data interpretation in these areas. Furthermore, only advertisements from the years 2020 and 2021 were analyzed, and these years were impacted by the COVID-19 pandemic. Often, it was impossible to decide whether a product is a drug or a dietary supplement.

### Suggestions for future studies and conclusions

The study showed that drug advertisements changed during the COVID-19 pandemic and rapidly responded to customer complaints. Overall, mostly psychological factors and marketing rather than pharmacological content dominated the advertisements. It also became evident that drug companies intentionally use the “soft” (ambiguous) wording of the Therapeutic Products Advertising Act to increase the impact of their financial investment. It is very evident that the regulations of the Therapeutic Products Advertising Act are only poorly controlled, resulting in several clear violations and many potential violations. Accordingly, the authors propose that the regulations of the Therapeutic Products Advertising Act be controlled more strictly by federal authorities to avoid misinformation of customers. Also, binding templates for the design of drug advertisements should be developed. A clear distinction must be made between advertisements for drugs and dietary supplements (Trabert and Seifert 2023). Ultimately, such clearly defined templates for drug advertisements will benefit the consumer and will make product comparison for them easier.

In future studies, additional years of the Apotheken Umschau should be studied. Going back to as far to the 1950s will provide valuable insights into the ways (west-)German society is dealing with drugs now and then and will also showcase how pharmacology developed as a discipline. The societal picture presented in current Apotheken Umschau issues clearly has much more similarities with the reality of (west-)German society of the 1950s than of the 2020s.

Along the same line, it will be most important to analyze television magazines for drug advertisements. Such magazines, targeted to older persons as well like Apotheken Umschau, also contain many drug advertisements. It will be very interesting to reveal how advertisements in television magazines and Apotheken Umschau differ from each other since television magazines do not have the “pharmacology” focus of Apotheken Umschau. It may be speculated that the pharmacological quality of advertisements in television magazines is even worse than in Apotheken Umschau because scientific quality control mechanisms are most likely to be poorer. It should also be noted that television itself is an important channel for drug advertisements. Therefore, such advertisements must be studied as well.

The study paradigm presented in our current paper can be applied to drug advertisement mechanisms in other countries. As a result of globalization, it will become more and more important to understand how different cultures conduct pharmacotherapy, not only at the professional level but also at the self-medication level. The analysis of drug advertisements in magazines is an excellent starting point for such studies. The analysis of the format of drug advertisements in countries with different pharmacotherapy cultures such as Germany, Norway, the USA, and Japan will be very informative.

Given the poor compliance to legal requirements of advertisements from the pharmacological point of view, it will be important to collaborate with expert lawyers to assess the exact extent to which advertisements comply with the law. In this context, it will also be important to examine legal compliance of advertisements in professional journals for medical doctors. Lastly, interdisciplinary collaboration with psychologists and marketing experts on the analysis of the mechanisms and effects on customers of drug advertisements is important. All these studies will ultimately result in better and scientifically founded drug advertisements and improve drug safety.

From the results of this study, it is clear that drug safety has no priority for the companies advertising their products because even basic statements such as “Für Risiken und Nebenwirkungen fragen Sie Ihren Arzt oder Apotheker oder lesen Sie die Packungsbeilage” are missing in more than 50% of the products studied and specific adverse effects are almost never provided. So, consumers may assume that the products advertised are not “real drugs” and do not report the consumption of these to their doctors. They may assume that the products are actually more like dietary supplements. However, it is well-known that non-prescription drugs and dietary supplements can have serious adverse effects (Taylor and Davies 2018; McCrae et al. 2018) and show dangerous interactions with prescription drugs (Parvez and Rishi 2019; Scherf-Clavel 2022). Thus, implementing strict regulations for drug advertisements for the public is an important contribution to improving drug safety. Medical doctors should consider the results of the present analysis when discussing drug therapy plans with their patients and ask for their self-medication as a result of advertisements in health magazines.

### Take-home messages

The present analysis of Apotheken Umschau revealed a number of important problems for consumer safety and drug safety that must be addressed:

- Drug advertisements rapidly respond to market demands.
- Drug advertisements may mislead and misinform consumers.
- Pharmacological evidence plays only a minor role in drug advertisements.
- Instead, psychology and marketing dominate the drug advertisements.
- An outdated societal picture is presented.
- Important aspects of the Therapeutic Products Advertising Act in Germany are not implemented.
- The actual costs of the advertised products remain elusive in most cases. Probably, the products are expensive.
- The public must be educated about the deficits in current drug advertisement practices.

### Supplementary Information

Below is the link to the electronic supplementary material.

Supplementary file1 (DOCX 2953 KB)

## Data Availability

All source data for this study are available upon reasonable request.

## Abbreviations

AVR: Arzneiverordnungsreport (drug prescription report)
DDD: Daily defined dose
INN: International Nonproprietary Name
