# Supplementary material for: Drug advertising in the German free-of-charge health magazine Apotheken Umschau (Pharmacy review): a critical analysis
Source: Naunyn Schmiedebergs Arch Pharmacol. 2023 Oct 4;397(4):2145–57. doi: 10.1007/s00210-023-02744-7 (PMC10933185; doi:10.1007/s00210-023-02744-7)
Supplement: Supplementary file 1 — Supplementary file1 (DOCX 2953 KB) [file 210_2023_2744_MOESM1_ESM.docx]

**Supplemental Figures S 1-S 11**

**Laura Keuper and Roland Seifert**

# Drug advertising in the German free-of-charge health magazine Apotheken Umschau (Pharmacy Review): A critical analysis

**Fig. S 1: Overview of the printed persons, shown in a bar chart.**

**Fig. S 2: Emotions shown, presented in a pie chart, where the emotion happiness is blue, no emotion is orange, pain is grey, and happiness and pain are yellow.**

**Fig. S 3: Persons in motion, shown in a pie chart.**

**Fig. S 4a: Approximate age of the printed Fig. S 4b: Ethnicity of the imprinted persons,**

**persons, represented in a pie chart, in which represented in a pie chart.**

**the age class over 40 years is blue, over 60**

**years orange, over 40 years grey and the**

**category child yellow.**

**Fig. S 5: Printed body part in focus, shown in a pie chart.**

**Fig. S 6: Printed nature motifs, shown in a pie chart.**

**Fig. S 7: Association of preparation name on complaints to be alleviated, where the category suggestive message is blue, neutral is orange and INN (international nonproprietary name) is grey.**

**Fig. S 8: Printed pack/tablet, shown in a pie chart.**

**Fig. S 9: Thematically appropriate editorial texts next to the advertisements, shown in a pie chart.**

**Fig. S 10: Colour design of the advertising statements, shown in a pie chart, where the respective colour also represents the colour in the diagram.**

**Fig. S 11a: Size of the mandatory Fig. S 11b: Colour readability Fig. S 11c: Colour of the text,**

**text in comparison to the ad of the mandatory text, represented in a pie chart.**

**text, represented in a pie chart. represented in a pie chart.**
